# Reduced Uteroplacental Perfusion Pressure (RUPP) causes altered trophoblast differentiation and pericyte reduction in the mouse placenta labyrinth

**DOI:** 10.1038/s41598-018-35606-x

**Published:** 2018-11-21

**Authors:** Bryony V. Natale, Prutha Mehta, Priscilla Vu, Christina Schweitzer, Katarina Gustin, Ramie Kotadia, David R. C. Natale

**Affiliations:** 1 Department of Comparative Biology and Experimental Medicine, Faculty of Veterinary Medicine, University of Calgary, Calgary, AB T2N4N1 Canada; 2 Department of Obstetrics and Gynecology in Reproductive Sciences, Faculty of Medicine, University of California San Diego, La Jolla, CA 92093 USA

## Abstract

This study characterized the effect of the reduced utero-placental perfusion pressure (RUPP) model of placental insufficiency on placental morphology and trophoblast differentiation at mid-late gestation (E14.5). Altered trophoblast proliferation, reduced syncytiotrophoblast gene expression, increased numbers of sinusoidal trophoblast giant cells, decreased *Vegfa* and decreased pericyte presence in the labyrinth were observed in addition to changes in maternal blood spaces, the fetal capillary network and reduced fetal weight. Further, the junctional zone was characterized by reduced spongiotrophoblast and glycogen trophoblast with increased trophoblast giant cells. Increased *Hif-1α* and TGF-β-3 *in vivo* with supporting hypoxia studies in trophoblast stem (TS) cells *in vitro*, support hypoxia as a contributing factor to the RUPP placenta phenotype. Together, this study identifies altered cell populations within the placenta that may contribute to the phenotype, and thus support the use of RUPP in the mouse as a model of placenta insufficiency. As such, this model in the mouse provides a valuable tool for understanding the phenotypes resulting from genetic manipulation of isolated cell populations to further understand the etiology of placenta insufficiency and fetal growth restriction. Further this study identifies a novel relationship between placental insufficiency and pericyte depletion in the labyrinth layer.

## Introduction

Preeclampsia is a human, pregnancy-related syndrome with clinical symptoms that include hypertension, proteinuria and in severe cases, risk of fetal and maternal mortality. Currently, there is no genetic screening and few biomarkers that reliably predict the disease, and its etiology is unknown. Poor placentation and reduced utero-placental perfusion are thought to contribute to preeclampsia, though whether poor placentation is the cause or consequence is debated. Human placental assessment is limited to observational studies or is restricted to placental tissue available from either early gestation or following delivery of the baby. As such, studying mid-gestation placentae affected by preeclampsia remains a challenge. Human and animal studies complement each other and move our understanding of preeclampsia and fetal growth restriction forward and as such, observations made in human studies lead to hypotheses that are tested in animal experiments, further refined using multiple animal models and are then retested in humans. It is within this cycle of preeclampsia and growth restriction investigation that this study falls. The symptoms that identify an ideal model of preeclampsia include hypertension, proteinuria, poor trophoblast invasion and endothelial damage. There are several well-characterized animal models of preeclampsia, and their merit has been debated and reviewed^1,2^. Reduced placental perfusion is thought to contribute to the development of preeclampsia^3^, resulting in the development of the reduced utero-placental perfusion pressure (RUPP) model^4^, in which there is an ~40% reduction in blood flow to the placentae. The model, first developed in the rat^4^ causes both maternal and fetal pathology including: increased mean arterial blood pressure, decreased glomerular filtration rate^5,6^, increased peripheral resistance, decreased cardiac index^7^, decreased renal pressure natriuresis and plasma flow, proteinuria, endothelial dysfunction^8^ and fetal growth restriction^9^. In addition, this model is associated with an imbalance of angiogenic factors including increased soluble (s)Flt-1, decreased vascular endothelial growth factor (VEGF) and placental growth factor (PLGF)^6,9,10^ and increased soluble endoglin (sENG) and placental hypoxia inducible factor-1 alpha (Hif1-*α*) with a reduction in heme oxygenase 1 expression (HO-1)^11^. Placental-derived mediators that act on the maternal endothelium have been proposed as a cause of preeclampsia^12^; supporting this, plasma from RUPP rats induces endothelial dysfunction in healthy pregnant vessels^8^. The RUPP model has been adapted to the mouse^13–15^, where it also results in increased maternal blood pressure^13,14^, increased serum sFlt-1^13^, increased maternal urinary albumin^14^, endotheliosis^14^, mesangial expansion^14^ and decreased fetal weight^13,14^. In order to further understand the placental pathology associated with RUPP, this study aimed to identify the changes within the layers and cell populations that contribute to the placenta, and the timing of these changes with respect to gestation and onset of RUPP.

## Results

### RUPP pregnancies result in fetal growth restriction, increased placental weight and altered labyrinth to junctional zone ratio

**Figure 1:**
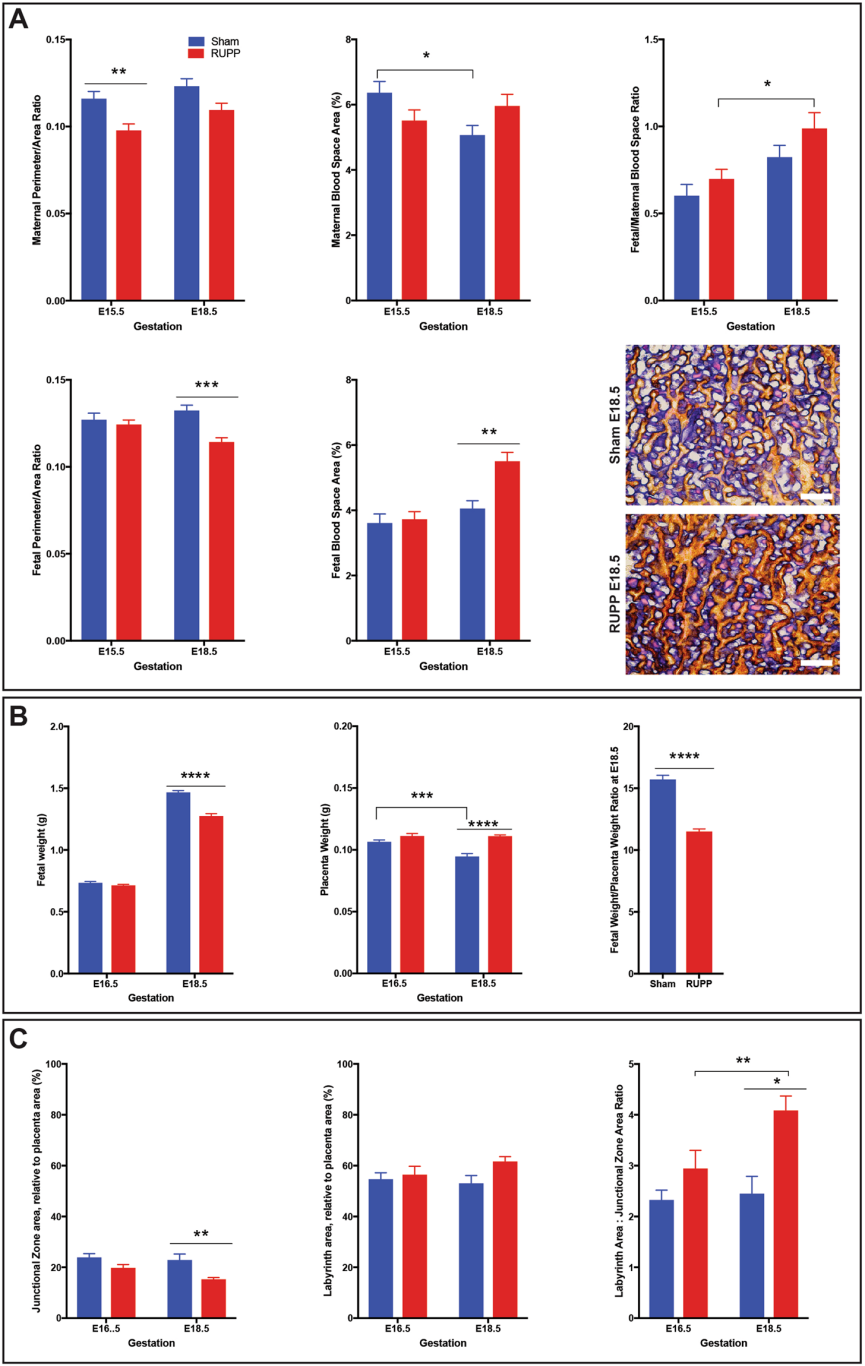
RUPP causes changes in placental structure and subsequently, altered placental and fetal weights. (**A**) Maternal and Fetal blood spaces and perimeter to area ratios are changed in response to RUPP at individual time points and between E16.5 and E18.5. Dual Iso-Lectin staining (fetal) and Alkaline Phosphatase staining (maternal) provides a visual of the relationship between maternal and fetal blood spaces in the placenta labyrinth (n ≥ 9 per treatment/gestational age). (**B**) Fetal and Placenta weights and their ratio are altered in response to RUPP (n ≥ 28 per treatment group/gestational age). (**C**) Junctional zone area is reduced in the RUPP placentae, altering the labyrinth/junctional zone ratio (n = 5 placentae per treatment/gestational age). Solid line with star indicates statistical significance between the treatment groups at the identified gestational age; bracket with star indicates statistical significance between the gestational ages of the identified treatment group; *p ≤ 0.05, **p ≤ 0.01, ***p ≤ 0.001, ****p ≤ 0.0001. Scale bar = 100 um.

To determine the effect of RUPP on placental development and fetal weight, assessments were made at two gestational ages, E15.5 and E18.5. Statistical analysis by two-way ANOVA addressed whether there was a difference between the Sham and RUPP group at each of the gestational ages, and whether there was a difference in response between the two gestational ages. The placenta develops to facilitate exchange of nutrients and waste and as such analysis included the fetal capillary network and maternal blood sinusoids within the labyrinth layer (herein referred to as fetal and maternal blood spaces, respectively^16–20^). This assessment included: area of blood spaces relative to total placental area; change in maternal and/or fetal blood space; maternal to fetal blood space ratio and the perimeter to area ratio, as an indicator of nutrient exchange. When compared to Sham operated, RUPP placentae did not have significantly altered maternal blood space at either E15.5 or E18.5, though between the two time points, there was a significant difference where the Sham placentae saw a decrease (p = 0.0173) in maternal blood space area between E15.5 and E18.5, while in the RUPP placentae, the relative maternal area remained the same (Fig. 1A). In response to RUPP, the maternal perimeter to area ratio was decreased at E15.5 (p = 0.0064; Fig. 1A). Fetal blood space did not change until E18.5 when both area and perimeter to area ratio were altered, with area increased (p = 0.0014), and perimeter to area ratio decreased (p = 0.0003; Fig. 1A), suggesting the potential for less efficient nutrient exchange in the RUPP placentae. In the Sham placentae relative fetal blood space area remained constant, with decreased maternal blood space area, while conversely, the RUPP saw increased relative fetal blood space area with constant maternal blood space area, which contributed to an increased (p = 0.0161) fetal/maternal blood space ratio across gestation in the RUPP placentae (Fig. 1A). As the fetal blood space area was not altered at E15.5, all remaining assessment was performed at E16.5 and E18.5 to allow 48hrs and 96hrs of reduced perfusion prior to assessment.

RUPP surgery performed at E14.5 resulted in reduced fetal weight (p < 0.0001) and increased placentae weight (p < 0.0001) at E18.5 (Fig. 1B). Sham placentae weight decreased (p = 0.0004) between E16.5 and E18.5, while the RUPP placenta weights were not altered (Fig. 1B). As an indicator of placental efficiency, fetal weight (FW) to placental weight (PW) ratio at E18.5 was assessed, with E18.5 RUPP pregnancies showing a decreased ratio when compared with Sham pregnancies (Fig. 1B)^21^. Litter size and resorption, of Sham and RUPP pregnancies, were assessed, with 15 Sham and 16 RUPP litters evaluated. Sham pregnancies had an average of 13.53 pups with 0.73 dead or resorbed, while RUPP pregnancies had 13.94 pups with 1.31 dead or resorbed. There was no statistical difference between the populations (Supplemental Fig. 1).

The area of each placental layer, measured relative to the total placental area, showed no change in the relative area of the labyrinth layer, though the junctional zone area was reduced (p = 0.0066) in response to RUPP at E18.5 (Fig. 1C). The ratio between the labyrinth and the junctional zone was increased (p = 0.0319) between E16.5 and E18.5 in response to RUPP, while the ratio in the Sham placentae remained constant and as such there was a significant ratio increase (p = 0.0028) in the RUPP at E18.5 (Fig. 1C)^22,23^.

### Microarray analysis of RUPP placentae

Genome-wide expression profiling was conducted by microarray to compare E16.5 Sham and RUPP placentae. 487 genes were affected (Fc > 1.25; P < 0.05), with 339 up regulated and 148 down regulated in the RUPP placentae (Supplemental Table 1). Microarray experiments were conducted on whole placenta at E16.5 to identify pathways and clusters that differed between the treatment groups. E16.5 was chosen, as the focus was on genes, clusters and pathways that were altered shortly after treatment and may contribute to the fetal/placenta phenotype. It is acknowledged that microarray on the whole placenta includes a mix of tissues including trophoblast, pericyte and endothelial, however as trophoblast cells have a known interaction with endothelial cells and possibly with pericytes, assessing the entire placenta was critical. Using Metascape (http://metascape.org)^24^, 389 unique genes and the top 20 clusters were identified (Table 1.). Studies including histology and/or qPCR at both E16.5 and E18.5, were performed to confirm microarray results and included the following placental components and their respective pathways: uNK cells- granzyme-mediated apoptotic signaling pathway, blood vessel morphogenesis and extracellular matrix organization and are highlighted in results described below. This assessment considered whether a change identified at E16.5 was still present at E18.5. Histological assessment provided the advantage of identifying where in the placenta the change was taking place.

**Table 1:** Top 20 altered gene clusters identified in RUPP placentae.

GO	Description	Count	%	Log10(P)
GO:0008626	granzyme-mediated apoptotic signaling pathway	6	1.63	−8.04
GO:0050921	positive regulation of chemotaxis	10	2.72	−4.43
GO:0048514	blood vessel morphogenesis	22	5.99	−4.21
GO:0061448	connective tissue development	12	3.27	−3.37
R-MMU-375276	Peptide ligand-binding receptors	10	2.72	−3.18
GO:1902414	protein localization to cell junction	3	0.82	−3.18
GO:0030449	regulation of complement activation	3	0.82	−3.07
GO:0043279	response to alkaloid	6	1.63	−2.93
GO:0006721	terpenoid metabolic process	5	1.36	−2.83
GO:0042461	photoreceptor cell development	5	1.36	−2.73
GO:0035313	wound healing, spreading of epidermal cells	3	0.82	−2.72
GO:1990542	mitochondrial transmembrane transport	5	1.36	−2.63
GO:0006081	cellular aldehyde metabolic process	5	1.36	−2.46
R-MMU-1474244	Extracellular matrix organization	11	3	−2.45
GO:0070374	positive regulation of ERK1 and ERK2 cascade	10	2.72	−2.43
GO:0042552	myelination	7	1.91	−2.3
GO:0010942	positive regulation of cell death	19	5.18	−2.28
GO:0001894	tissue homeostasis	9	2.45	−2.27
GO:0072678	T cell migration	4	1.09	−2.24
GO:0032870	cellular response to hormone stimulus	16	4.36	−2.23

Gene set enrichment analysis of microarray results comparing E16.5 Sham with E16.5 RUPP placentae was conducted using Metascape (http://metascape.org). Top 20 clusters with their representative enriched terms. Count represents the number of genes with p < 0.05 and absolute FC > 1.25 with membership in the given ontology term. % represents the percentage of total genes with p < 0.05 and absolute FC > 1.25 that are found in the given ontology term. Log10(P) is the p-value in log base 10. GO = GO Biological Processes; R-MMU = Reactome Gene Sets.

### RUPP placentae have increased HIF-1α with altered proliferation and differentiation of labyrinth trophoblast

**Figure 2:**
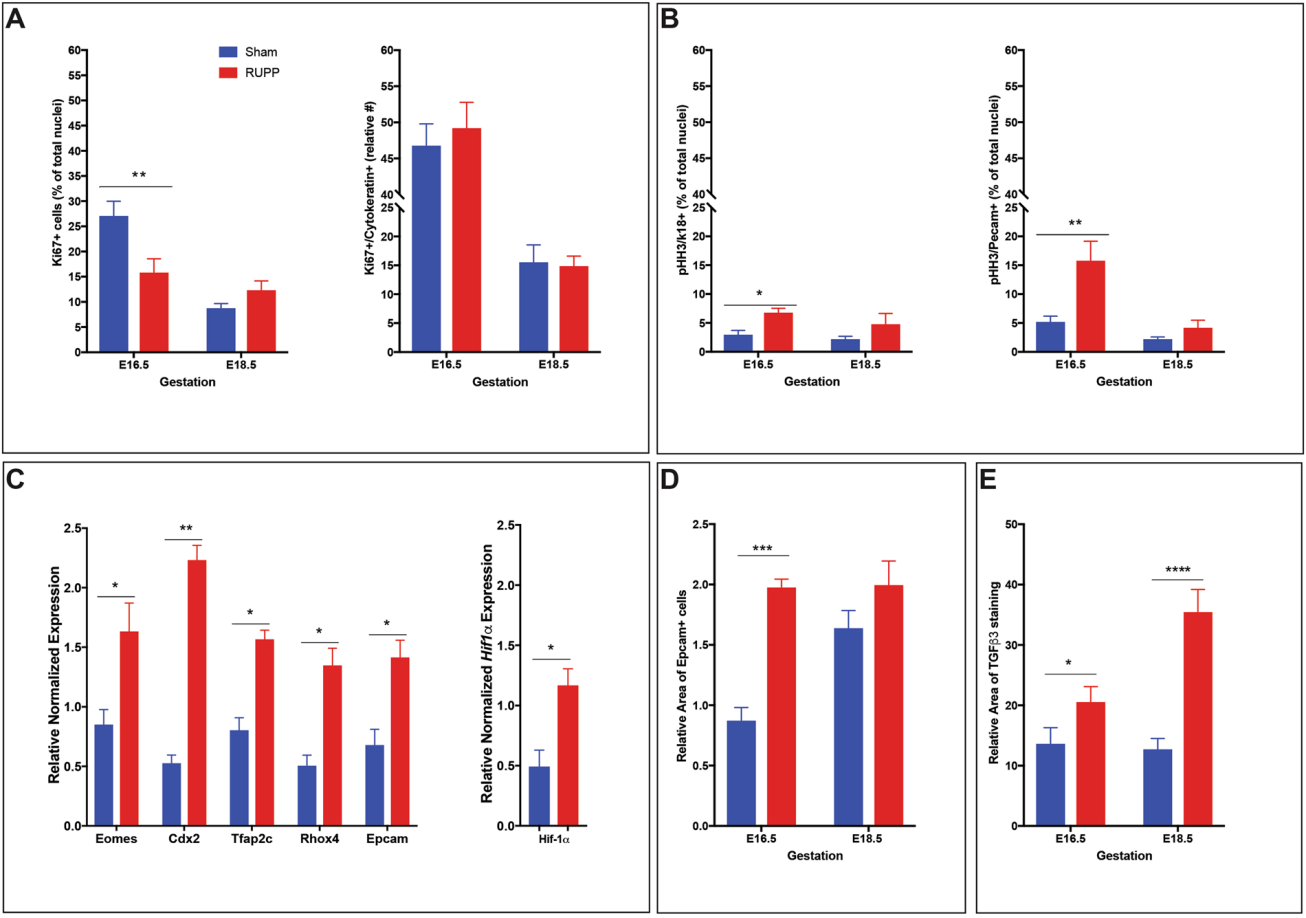
RUPP creates hypoxic placenta environment with altered proliferation. (**A**) Total Ki67 (n = 12 placentae per treatment/gestational age) and trophoblast specific Ki67 (n = 9 placentae per treatment/gestational age) and (**B**) pHH3^+^ trophoblast (k18) and pHH3+ fetal endothelial (Pecam) as measures of relative placental proliferation (n = 5 placentae per treatment/gestational age). (**C**) Evaluation of hypoxia (*Hif-1α*), TS cell populations (*Eomes, Cdx2* and *Tfap2c*) and progenitor populations (*Rhox4* and *Epcam*) in the E16.5 placentae by qPCR (n = 3 placentae per treatment). (**D**) Evaluation of the Epcam^+^ progenitor population by IHC (n = 3 placentae per treatment/gestational age). (**E**) Evaluation of hypoxia (TGF-β-3) by IHC (n ≥ 3 placentae per treatment/gestational age). Stars indicate statistical significance between the treatment groups at the identified gestational age: *p ≤ 0.05, **p ≤ 0.01, ***p ≤ 0.001, ****p ≤ 0.0001.

**Figure 3:**
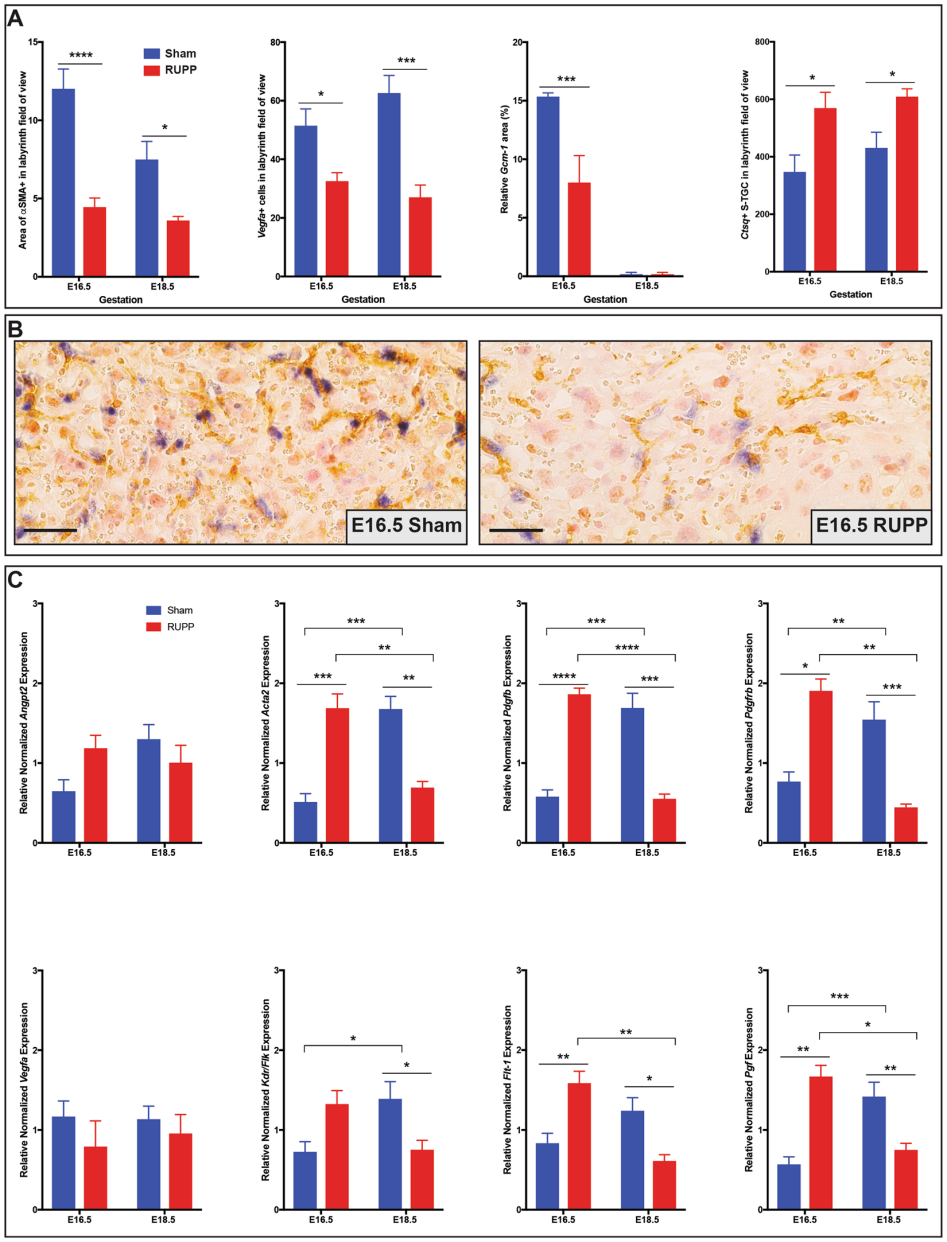
RUPP placentae have altered trophoblast differentiation, reduced pericytes and altered expression of genes associated with blood vessel formation. (**A**) RUPP placentae see reduced pericyte (αSMA; (n ≥ 5 placentae per treatment/gestational age) and Syn-T (*Gcm-1;* (n ≥ 3 placentae per treatment/gestational age) populations, with increased S-TGC (*Ctsq;* (n ≥ 5 placentae per treatment/gestational age) populations, and decreased *Vegfa*^+^ cells (n ≥ 7 placentae per treatment/gestational age) in the labyrinth. (**B**) Dual pericyte (αSMA)/*Vegfa* staining, identifying reduction of both populations in response to RUPP at E16.5. (**C**) Assessment of genes associated with blood vessel formation by qPCR (n = 3 placentae per treatment/gestational age). Solid line with star indicates statistical significance between the treatment groups at the identified gestational age; bracket with star indicates statistical significance between the gestational ages of the identified treatment group; *p ≤ 0.05, **p ≤ 0.01, ***p ≤ 0.001, ****p ≤ 0.0001. Scale bar = 25 um.

Analysis of cell proliferation throughout the placenta in response to RUPP treatment was assessed by Ki67 and pHH3 immunohistochemistry (IHC): Ki67 identified proliferation of all cell types; Ki67/cytokeratin and pHH3/K18 were used to identify proliferating labyrinth trophoblast while pHH3/Pecam was used to identify proliferating labyrinth endothelial cells. Total Ki67 staining identified decreased (p = 0.0018) total proliferation at E16.5 in response to RUPP (Fig. 2A). Sham placentae had a significant decrease (p < 0.0001) in proliferation between E16.5 and E18.5, while the RUPP placentae did not (Fig. 2A). Dual Ki67/cytokeratin staining showed trophoblast cell proliferation in the labyrinth was not altered in response to RUPP (Fig. 3B). pHH3^+^/K18^+^ dual staining however, showed increased (p = 0.0493) trophoblast proliferation in the labyrinth, in response to RUPP at E16.5, with a non-significant increase at E18.5 (Fig. 2B)^15^. pHH3^+^/Pecam^+^ dual staining showed increased (p = 0.0020) endothelial proliferation in the labyrinth, in response to RUPP at E16.5, and the increase was similarly, not significant at E18.5 (Fig. 2B). Trophoblast stem (TS) cell marker *Eomes* and labyrinth progenitor marker *Rhox4b*^25^ were up-regulated in the microarray and confirmed by qPCR (p = 0.0434 and p = 0.0219 respectively; Fig. 2C). Also, significantly increased in the E16.5 RUPP placenta, were TS cell markers *Cdx2* (p = 0.0013) and *Tfap2c* (p = 0.016), and labyrinth progenitor marker *Epcam* (p = 0.0382), when assessed by qPCR (Fig. 2C). Histological assessment by IHC further supported increased Epcam expression at E16.5 (p = 0.0010), however at E18.5 there was no difference between the Sham and RUPP placentae (Fig. 2D).

Analysis of microarray data identified genes associated with a hypoxic placenta environment with the up regulation of *Adam8*, *Pdgfb* and the down regulation of *Ednra* (Supplemental Table 1^26^). As such oxygen sensitive hypoxia inducible factor-1a (*Hif-1α)* and its downstream effector TGF-β-3 were assessed as a measure of a hypoxic environment. *Hif-1α* expression was significantly increased at E16.5 (p = 0.0252) when assessed by qPCR (Fig. 2C). TGF-β-3 as a downstream target of HIF-1*α* is shown to be elevated when Hif-1*α* expression is increased^27^. Area of TGF-β-3 expression in response to RUPP was increased at E16.5 (p = 0.0201) and at E18.5 (p < 0.0001) and also increased between the two time points (p < 0.0001; Fig. 2E).

### RUPP placentae have a reduction in pericytes and decreased expression of *Vegfa*

**Figure 4:**
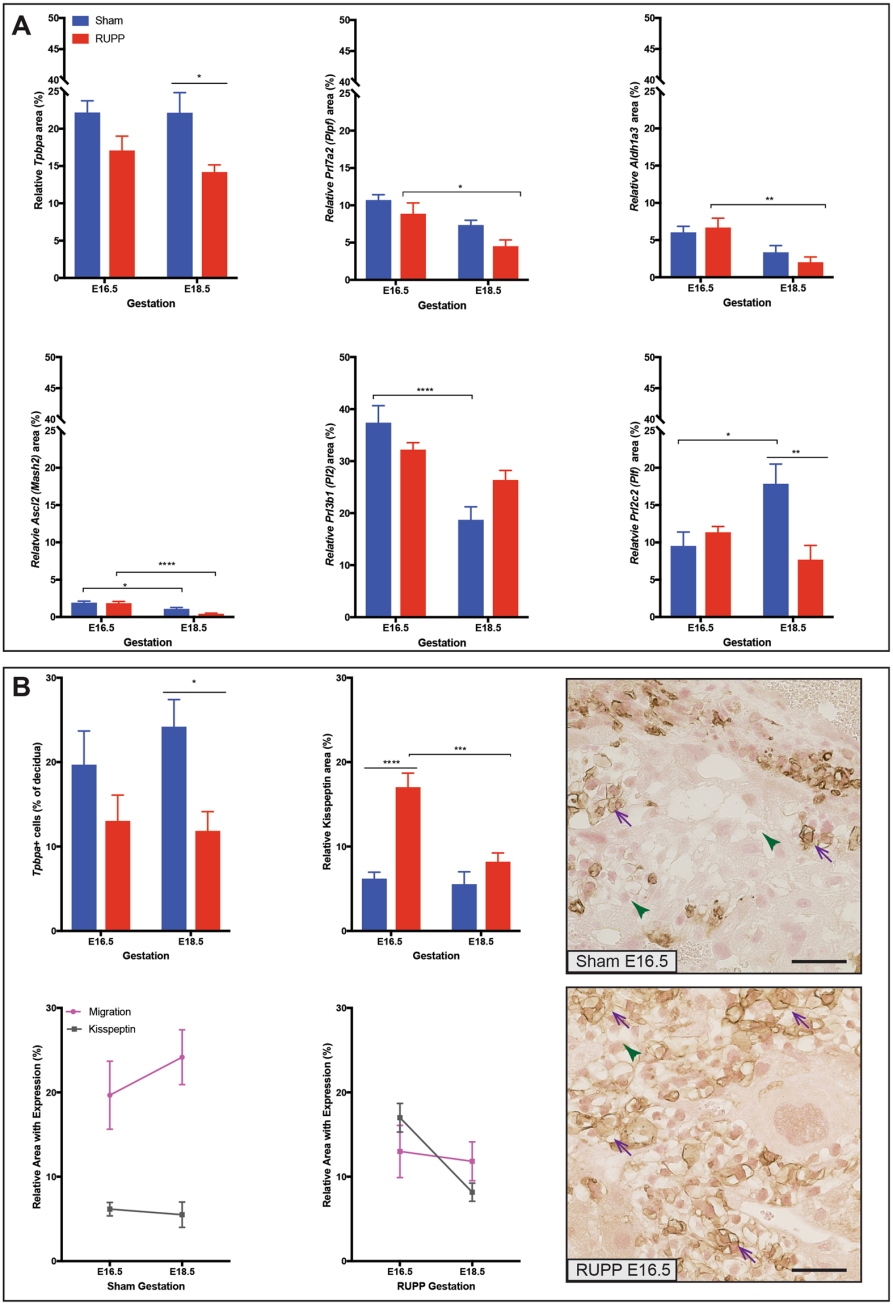
Junctional Zone trophoblast sub-types are altered in response to RUPP, with reduced migration. (**A**) Trophoblast populations of the junctional zone are altered by RUPP as assessed by ISH (*Tpbpa, Prl7a2, Aldh1a3, Prl3b1* and *Prl2c2* n = 6; *Ascl2* n = 9). (**B**) Trophoblast migration into the decidua as assessed by *Tpbpa*^+^ staining within the decidua is reduced in response to RUPP (n = 6) and Kisspeptin^+^ staining (n = 6) is increased. There is an inverse relationship between migration and Kisspeptin expression. Kisspeptin expression is restricted primarily to the Gly-T cells (arrows are Kiss^+^ Gly-T; arrowheads are Kiss^−^ Gly-T). Solid line with star indicates statistical significance between the treatment groups at the identified gestational age; bracket with star indicates statistical significance between the gestational ages of the identified treatment group; *p ≤ 0.05, **p ≤ 0.01, ***p ≤ 0.001, ****p ≤ 0.0001. Scale bar = 25 um.

*α*SMA was used as a marker of pericytes in the placenta labyrinth and IHC identified a reduction in relative *α*SMA^+^ area (pericytes) in the E16.5 labyrinth (p < 0.0001) and at E18.5 (p = 0.0214) in in response to RUPP (Fig. 4A,B). With more than double the *α*SMA^+^ area, Sham placentas saw the pericyte area decrease (p = 0.0044) between E16.5 and E18.5, while the RUPP placentas did not. *Gcm-1*, expressed in syncytiotrophoblast (Syn-T), was assessed by *in situ* hybridization (ISH) and showed a reduction in relative staining area (p = 0.0001) at E16.5 in the RUPP placentae, when compared to the Sham placentae (Fig. 3A). Of note, while *Gcm-1* area was reduced at E18.5, it has been published that its expression in Syn-T decreases after E17.5^28^, and as such little to no staining at E18.5 was expected. The number of sinusoidal trophoblast giant cells (S-TGC) evaluated by *Ctsq* expression^29^, saw an increase in response to RUPP when compared to Sham at E16.5 (p = 0.0102), and at E18.5 (p = 0.0499; Fig. 3A). Labyrinth-specific, *Vegfa* expression as measured by the number of *Vegfa*^+^ cells in the labyrinth was decreased at E16.5 (p = 0.0121) and at E18.5, (p = 0.0001) in response to RUPP (Fig. 3A,B). Combined with altered fetal blood space area and perimeter/area ratio at E18.5 with a reduction in labyrinth pericytes and *Vegfa*^+^ cells, we looked at markers of angiogenesis in the whole placenta, by qPCR. There was an interesting pattern observed in that four of these genes, *Acta2, Pdgfb, Pdgfrb* and *Pgf*, were significantly increased at E16.5 in response to RUPP (p = 0.0006; p < 0.0001; p = 0.0013; p = 0.0007, respectively), with a significant reduction at E18.5 (p = 0.0019; p = 0.0002; p = 0.0017; p = 0.0140, respectively), while conversely, in the Sham placentae, their expression significantly increased between E16.5 and E18.5 (p = 0.0006; p = 0.0002; p = 0.0126; p = 0.0036, respectively; Fig. 3C). Analysis of the Vegfa receptors showed Sham placentae had a significant increase in Vegfa receptor-2, *Flk (Kdr)* expression between E16.5 and E18.5 (p = 0.0412) with expression significantly reduced in the RUPP placentae at E18.5 (p = 0.0490; Fig. 3C). Vegfa receptor-1, *Flt-1* expression was significantly increased at E16.5 in response to RUPP (p = 0.0082), however at E18.5, expression in the RUPP placentae was significantly reduced (p = 0.0211; Fig. 3C). Contrary to the *Vegfa* expression analysis as measured by the number of *Vegfa*^+^ cells, the reduction observed in *Vegfa* expression as assessed by qPCR in the whole placenta was not significant, highlighting the importance of evaluating expression in the placenta by layer (Compare Fig. 3A with 3C). *Angpt2* expression was not significantly altered in response to RUPP or across gestation (Fig. 3C).

### In response to RUPP, junctional zone trophoblast marker expression is altered and migration to the decidua is reduced

In the junctional zone, the area of *Tpbpa* staining (junctional zone trophoblast subtypes) was reduced (p = 0.017) at E18.5 in the RUPP placentae (Fig. 4A). *Ascl2 (Mash2)*, a marker of junctional zone progenitors, saw a similar decrease in area in both Sham (p = 0.0168) and RUPP (p < 0.0001) placentae between E16.5 and E18.5 (Fig. 4A). *Prl7a2* (*Plpf;* spongiotrophoblast) area showed a similar trend in both RUPP and Sham placentae, though in the RUPP placentae, area was significantly decreased (p = 0.0119) between E16.5 and E18.5 (Fig. 4A). *Aldh1a3*, marker of glycogen trophoblast, was not significantly different between the two groups at either E16.5 or at E18.5, however in response to RUPP the reduction of the area of expression between E16.5 and E18.5 was statistically significant (p = 0.0056; Fig. 4A). *Prl2c2* (*Plf*; parietal TGC, spiral artery TGC and canal artery TGC) area was significantly altered when comparing RUPP placentae with Sham. At E16.5 the area was similar between the two groups, however at E18.5 area was decreased (p = 0.0028) in the RUPP placentae. Between E16.5 and E18.5 the Sham placentae saw an increased (p = 0.0129) area while the area in the RUPP placentae was reduced, though not significantly (Fig. 4A). *Prl3b1 (Pl2*; spongiotrophoblast, parietal TGC, spiral artery TGC and canal artery TGC) area decreased (p < 0.0001) between E16.5 and E18.5 in the Sham placentae, while in the RUPP placentae the area remained similar (Fig. 4A). The key differences noted were that Sham placentae had a decreased TGC population area relative to placenta size, with a constant spongiotrophoblast and Gly-T population, while RUPP placentae had a constant TGC population and a shrinking spongiotrophoblast and Gly-T population, relative to placenta size. We showed that Kisspeptin (Kiss-1) was localized primarily to the junctional zone and specifically the Gly-T (Fig. 4B), and its area was increased (p < 0.0001) at E16.5 in the RUPP placentae. *Tpbpa* positive cells migrate from the junctional zone to the decidua after E12.5^30^. Kiss-1 has been shown to inhibit human trophoblast migration *in vitro*^31^, and therefore, we looked for a similar pattern *in vivo*. The relative area of Kiss-1 expression did not change between E16.5 and E18.5 in the Sham placentae, while the increase at E16.5 in the RUPP meant a significant change (p = 0.0002) in area of expression between E16.5 and E18.5 (Fig. 4B). *Tpbpa*^+^ cells in the decidua are cells that have migrated from the junctional zone to the decidua. As such, they were evaluated as a measure of migration. Plotting *Tpbpa*^+^ migration with Kiss-1^+^ area identified that Sham decidua had increased migration and were associated with reduced Kiss-1^+^ area, while increased Kiss-1^+^ area was associated with less *Tpbpa*^+^ migration in the RUPP placentae (Fig. 4B). *Tpbpa*^+^ migration to the decidua was assessed in response to RUPP and was reduced (p = 0.0273).

**Figure 5:**
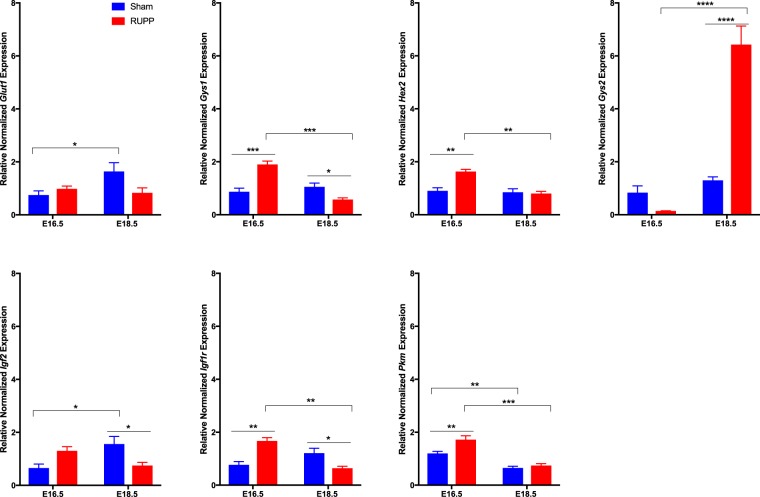
Expression of genes involved in glucose transport, metabolism and growth were altered in response to RUPP. RUPP placentae saw increased expression of *Gys1, Hex2, Igf1r* and *Pkm* at E16.5 with a significant decrease between E16.5 and E18.5. *Gys2* was increased at E18.5 while *Igf2* was decreased. Glut1 expression increased between E16.5 and E18.5 in Sham placentae but not in response to RUPP. Solid line with star indicates statistical significance between the treatment groups at the identified gestational age; bracket with star indicates statistical significance between the gestational ages of the identified treatment group, *p ≤ 0.05, **p ≤ 0.01, ***p ≤ 0.001, ****p ≤ 0.0001; (n = 3 placentae per treatment/gestational age).

In poorly functioning placentae, placental glycogen accumulation has been identified both in human placental pathologies and in animal models^30,32–34^. It is unknown whether this accumulation is due to limited ability to access glucose from the accumulated glycogen, thereby contributing to fetal growth restriction or whether the placenta compensates for increased maternal glucose and protects the fetus by limiting the glucose transport to the fetus. Glucose transporters in early pregnancy have been shown to have a capacity (maximal transport) that exceeds actual transport rates as the glucose concentration gradient has a greater effect on the regulation of glucose transport^35^. However, by late gestation, glucose transport capacity has a significant role in the regulation of transport to the fetus, with glucose concentration contributing limited glucose transport^35^. *Glut1* expression in the Sham placentae saw a significant increase between E16.5 and E18.5 (p = 0.0366), while the RUPP placentae did not, suggesting that glucose transport may be limited in the RUPP pregnancies (Fig. 5). At E16.5, the RUPP placentae had an increase in *Hex2* (p = 0.0023), which catalyzes the conversion of glucose to glucose-6-phosphate, the first step in glycolysis^36^ (Fig. 5); and an increase in *Pkm* (p = 0.0095; Fig. 5), a glycolytic enzyme^37^, however both drop off to the same levels as the RUPP by E18.5. Glucose-6-phosphate can mediate activation of Gys1 and Gys2^38^, playing a key role in controlling glycogen synthesis. Expression of *Gys1* was increased at E16.5 (p = 0.0006) and decreased at E18.5 (p = 0.0446) while *Gys2* was increased 5-fold in the RUPP placenta at E18.5 (p < 0.0001; Fig. 5). Igf2 acts primarily through the type-1 Igf receptor (Igf1r) in the placenta^39^. This binding mediates changes in cell proliferation and survival, metabolism and uptake of nutrients. At E16.5 *Igf1r* expression was increased in the RUPP placentae, however, at E18.5 both *Igf2* and *Igf1r* were decreased (Fig. 5). Together, this suggests that glucose metabolism is altered in the RUPP placentae.

Elevated levels of inflammatory cytokines are associated with preeclampsia, specifically; *IL-6* and *TNFa* are increased in the maternal circulation and the placenta^40,41^. As such, we evaluated expression of *IL-6* and *TNFa* in the Sham and RUPP placentae. While *TNFa* expression increased between E16.5 and E18.5, there was no difference in response to RUPP in expression of either *IL-6* or *TNFa* (Supplemental Fig. 2.). It is possible that there are other markers of inflammation that are altered within the model, however further assessment was not performed as there was no change to these two markers.

### Increased uNK cell populations in RUPP placentae

**Figure 6:**
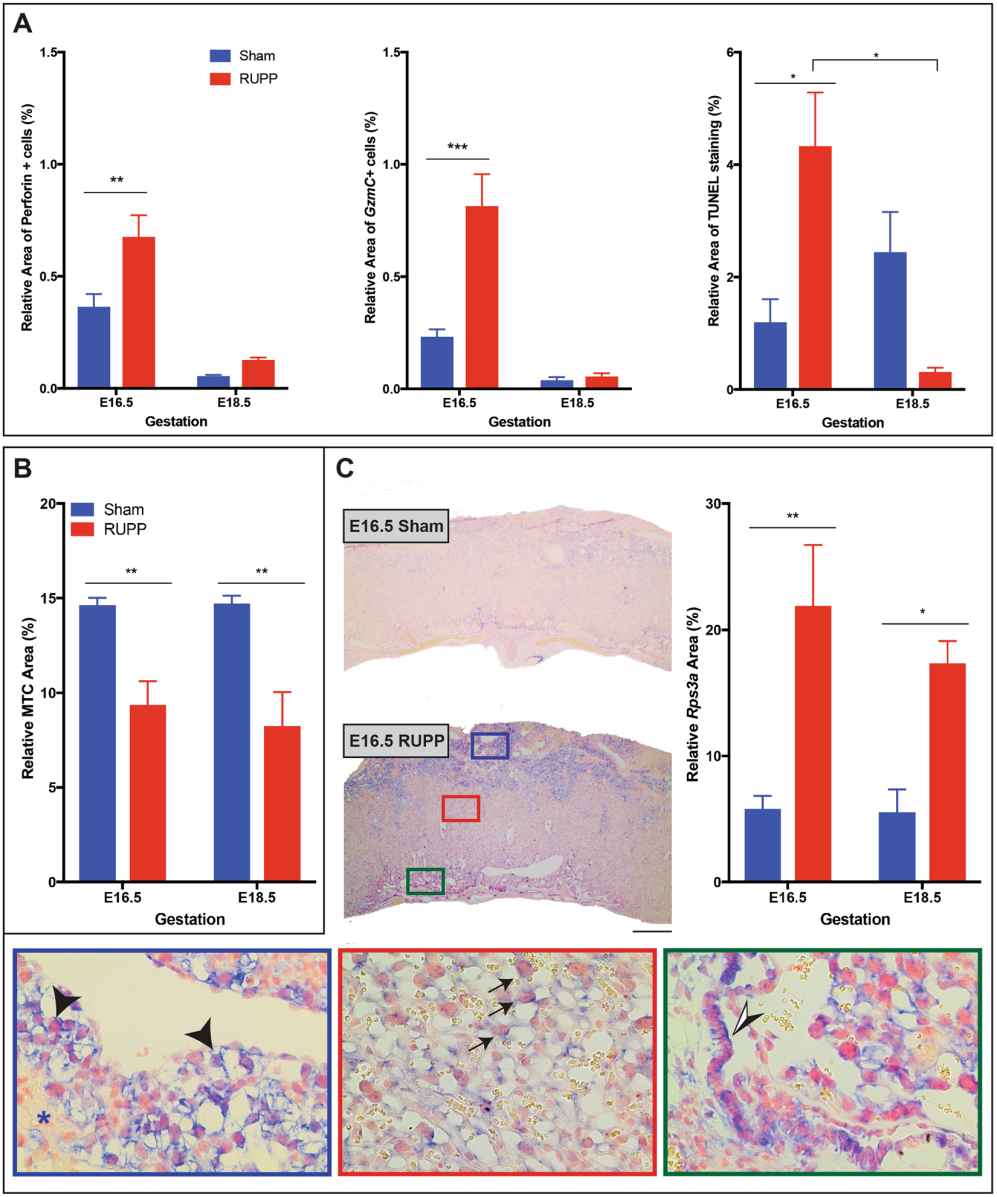
RUPP have increased uNK cell presence with increased TUNEL and altered placental ECM. (**A**) uNK cell expression of perforin (n ≥ 6) and *granzyme C (Gzmc*; n ≥ 4*)* were increased in RUPP placentae at E16.5 with an associated increase the relative area of TUNEL staining (n ≥ 3). (**B**) Masson’s Trichrome (MTC) staining was used to evaluate the ECM component collagen, which was significantly reduced in RUPP placentae (n = 5). (**C**) *Rps3a* ISH expression (n = 3) was increased in response to RUPP, ISH in the E16.5 placenta provides visual of difference in expression between Sham and RUPP placentae, inserts show staining by layer; blue inset identifies *Rps3a* in the Gly-T (black arrow head) and absence of *Rps3a* in the sp-T (black star); red inset identifies *Rps3a* in the S-TGCs of the labyrinth (black arrow identifies positive S-TGC); green inset identifies *Rps3a*^+^ cells in the chorion (black and white arrow head). Solid line with star indicates statistical significance between the treatment groups at the identified gestational age; bracket with star indicates statistical significance between the gestational ages of the identified treatment group, *p ≤ 0.05, **p ≤ 0.01, ***p ≤ 0.001, ****p ≤ 0.0001. Scale bar = 500 um.

Microarray data identified the granzyme-mediated apoptotic signaling pathway (GO:008626) as the most significantly altered pathway, with 6 up regulated genes. Additionally, there were 9 genes altered in the cytolysis: rupture of cell membranes and loss of cytoplasm pathway (GO:0019835); 8 genes up regulated, 1 down regulated. uNK assessment included perforin staining by IHC and *Gzmc* staining by ISH, showing a significant in increase at E16.5 in the area of perforin^+^ (p = 0.0036) and *Gzmc*^+^ cells (p = 0.0002), though at E18.5 there was no statistical difference between the two treatment groups, with the population decreased between E16.5 and E18.5 in both groups (Fig. 6A). Granzyme C protein expression is regulated by mRNA abundance^42^, is most closely related to human Granzyme H and can induce cell death^43^. TUNEL staining was localized primarily to the decidua, and at E16.5 TUNEL staining in the RUPP placentas was significantly increased (p = 0.0315; Fig. 6A), suggesting that the increased presence of perforin^+^ and *Gzmc*^+^ uNK cells may contribute to the increased cell death observed in the E16.5 RUPP placentae. At E18.5, TUNEL staining was non-significantly reduced in the RUPP placentae when compared with the Sham placentae, however the RUPP placentae had a significant reduction in TUNEL staining between E16.5 and E18.5 (p = 0.0315). If the perforin^+^ and *Gzmc*^+^ uNK cells do in fact contribute to the increased cell death observed at E16.5 in the RUPP placentae, then it would stand to reason that with the reduction of perforin^+^ and *Gzmc*^+^ uNK observed at E18.5, that there would be less cell death and thereby less TUNEL staining. This hypothesis would require further experimentation and while worthy of investigation is beyond the scope of the present study.

### RUPP induces structural and functional changes within the placenta

Hypoxia alters the deposition and degradation of collagen and other ECM molecules in tumor cells^44^. During angiogenesis, pericytes contribute to both the deposition and degradation of collagen, and in studies in which there was a decrease or absence of the pericyte marker, *NG2* collagen deposition was reduced^45^. As our data suggests placental hypoxia and a reduced population of pericytes, collagen was assessed by Masson’s Trichrome staining. As a percentage of placenta area, collagen area did not change across gestation in either Sham or RUPP placentae, however, in response to RUPP, collagen area was significantly reduced at both E16.5 (p = 0.0089) and E18.5 (p = 0.0018; Fig. 6B).

Ribosome protein expression is increased in actively proliferating cells and increased ribosome biogenesis is a feature of growth^46^. Ribosome synthesis supplies the translational machinery required to maintain protein production^47^, additionally, ribosomal proteins have been shown to have secondary functions^48^. Specifically, Ribosomal Protein 3 A (Rps3a) identified in the microarray, has physiological functions including a role in apoptosis^47,49^, cell transformation^50^, and initiation of translation^51^. Histological evaluation of *Rps3a* expression at E16.5 and E18.5, identified the junctional zone as the layer with the most staining- specifically Gly-T, with S-TGC’s in the labyrinth and the Epcam^+^ population in the chorion also staining positive (Fig. 6C). *Rps3a* area was significantly increased in response to RUPP at both E16.5 (p = 0.0068) and E18.5 (p = 0.330; Fig. 6C). Expression did not change across gestation in either treatment group.

### Hypoxia explains some, though not all trophoblast changes seen in the RUPP placentae

Both mouse and rat models of RUPP report elevated Hif-1*α*^11,14^ (Fig. 2), and therefore, TS cell proliferation and differentiation were evaluated in the context of hypoxia. TS cells in hypoxia have been studied^52–57^, as such TS hypoxia studies aimed to evaluate which trophoblast population changes (including TS, progenitor and differentiated trophoblast populations) paralleled the changes seen in the RUPP placentae. Additionally, downstream changes to markers of angiogenesis and hypoxia were considered and compared to the RUPP model. TS cells were cultured in normoxic (humidified air) and hypoxic (1% O_2_) air for 2, 4 and 6 days in both proliferating (in the presence of FGF4) and differentiating (in the absence of FGF4) conditions.

**Figure 7:**
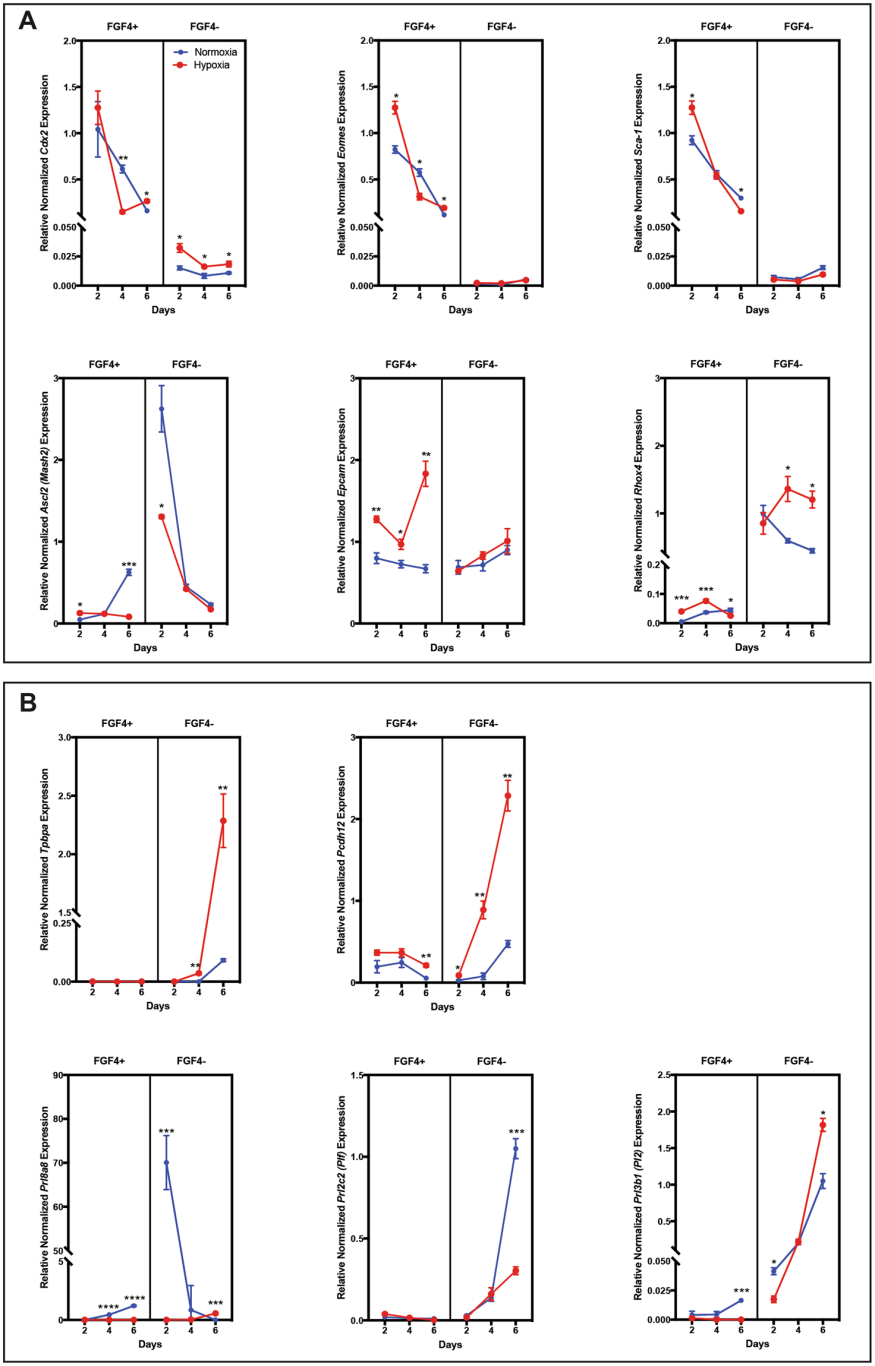
TS cells grown in hypoxic conditions have altered gene expression of TS, labyrinth progenitor and junctional zone markers. (**A**) Assessment of TS and progenitor markers by qPCR in TS cells grown in normoxic and hypoxic conditions in the presence and absence of FGF4. *Eomes, Sca-1, Epcam*, *Ascl2*, and *Rhox4* saw in initial increase in expression after 2 days in hypoxic +FGF4 conditions, while *Cdx2* and *Rhox4* saw an increase expression in hypoxia in the absence of FGF4. *Sca-1* and *Ascl2* highlight that the length of time in hypoxia can reduce expression levels. Results highlight that hypoxia alone does not universally alter TS and progenitor gene expression, rather the time in hypoxia or hypoxia combined with the presence or absence of growth factors alter gene expression. (**B**) Assessment of junctional zone trophoblast markers by qPCR in TS cells grown in normoxia and hypoxia in the presence and absence of FGF4. TS cells cultured in hypoxic conditions in the absence of FGF4 have increased expression of *Tpbpa, Pcdh12 and Prl3b1*, with reduced expression of *Prl2c2* and *Prl8a8*. Stars indicate statistical significance, *p ≤ 0.05, **p ≤ 0.01, ***p ≤ 0.001, ****p ≤ 0.0001 (n = 3 per treatment/day).

**Figure 8:**
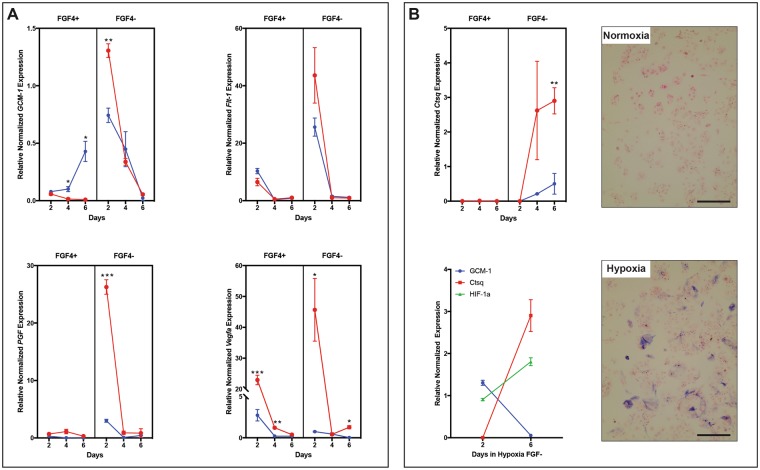
TS cells grown in hypoxic conditions see decreased GCM-1 expression as Hif-1α levels increase while the inverse occurs with Ctsq expression. (**A**) *Gcm-1* expression peaks on Day 2 in the presence of FGF4, in both normoxic and hypoxic conditions, with expression greater in hypoxia. In the absence of FGF4, the timing of peak expression of *Flt-1, Pgf* and *Vegfa* match that of *Gcm-1*, with expression greater in hypoxia. (**B**) As Hif-1α expression increases in hypoxic conditions, Gcm-1 (Syn-T) expression decreases and Ctsq (S-TGC) increases. Ctsq expression peaks on Day 6 in the absence of FGF4, with expression highest in hypoxic conditions. Alkaline Phosphatase staining identifies S-TGC differentiation of TS cells in normoxic and hypoxic conditions in the absence of FGF4. Stars indicate statistical significance, *p ≤ 0.05, **p ≤ 0.01, ***p ≤ 0.001, ****p ≤ 0.0001 (n = 3 per treatment/day). Scale bar = 100 um.

When comparing the hypoxic TS cell cultures with the trophoblast populations in the RUPP placentae there were several parallels. Looking at labyrinth trophoblast sub-populations, in proliferating conditions (with FGF4), TS cell, TS cell-like and progenitor markers, *Cdx2*, *Eomes, Sca-1, Epcam* and *Rhox4* were increased in hypoxia in at least one time point, supporting the hypothesis that hypoxia was a possible cause of the increased TS and trophoblast progenitor populations identified in the RUPP placentae (Fig. 7A). In hypoxic differentiating conditions (without FGF4), expression of *Ctsq*, a marker of S-TGC was increased, which was consistent with the increased *Ctsq*^+^ population seen in response to RUPP (Fig. 7A). The RUPP placentae displayed decreased *Gcm-1* expression; as such we looked at the effect of hypoxia on expression of *Gcm-1* and pro-angiogenic factors (*Pdgfb, Vegfa*), as these genes are known mediators of capillary development and pericyte recruitment. Peak *Gcm-1* expression had the same timing in both normoxia and hypoxia, however it was greater in hypoxic conditions (Fig. 8A), similarly to what has been previously published^58^. The pattern of expression of *Gcm-1* overlapped with *Vegfa* and *Pdgfb* expression in both conditions (Fig. 8A). As *Hif-1α* levels increased in culture, *Gcm-1* expression decreased (Fig. 8A) supporting the idea that hypoxia can induce Gcm-1 degredation^59^. It is worth noting, in hypoxic-differentiating culture conditions that as *Hif-1α* expression increased, *Gcm-1* expression decreased and *Ctsq* expression increased (Fig. 8B), which was a pattern similar to that seen in the RUPP placentae. TS cells in differentiating conditions were stained with alkaline phospatase, to identify S-TGCs, and there was an increased population in hypoxia when compared with normoxia (Fig. 8B), further supporting the concept that there is increased differentiation to S-TGC in hypoxia. In the junctional zone, there were both similarities and differences between hypoxic culture expression and RUPP placentae expression of trophoblast sub-types. Populations with similar patterns included spongiotrophoblast and TGCs. *Prl3b1* expression in TS cells was increased in hypoxic conditions (Fig. 7B), which may explain why the RUPP placentae did not see the same reduction between E16.5 and E18.5 as the Sham placentae. *Prl8a8* was used as a marker of Sp-T in the TS cells^60^ and was reduced in differentiating conditions in response to hypoxia, which mirrors the reduction in Sp-T area identified in the RUPP placentae (Fig. 7B). *Prl2c2 (Plf*) expression identifies TGCs and was similarly decreased in response to hypoxia (Fig. 7B); this too mirrors the area of expression profile seen in the RUPP placentae. Conversely, *Tpbpa, Ascl2* and *Pcdh12* expression in the hypoxic environment did not mirror expression patterns seen in the RUPP placenta. Where *Tpbpa* expression in TS cells was increased in response to a hypoxic environment (Fig. 7B), its area was decreased in response to RUPP. *Ascl2*, identifying junctional zone progenitors was not changed in the placenta, however the TS cell population saw a decrease in expression in response to hypoxia (Fig. 7B). The relative Gly-T population in the RUPP placenta decreased between E16.5 and E18.5 while TS cells in hypoxia saw increased expression of *Pcdh12* in hypoxic conditions (Fig. 7B). The results show that hypoxia alone does not make universal changes in gene expression, but rather hypoxia in the presence of FGF4 affects the expression patterns of some TS cell and progenitor markers, while in the absence of FGF4 hypoxia affects different expression patterns, including the markers of TGCs. Together this data supports a role for hypoxia as a contributing factor to some, though not all of the altered trophoblast populations identified in the RUPP placentae.

## Conclusion

**Figure 9:**
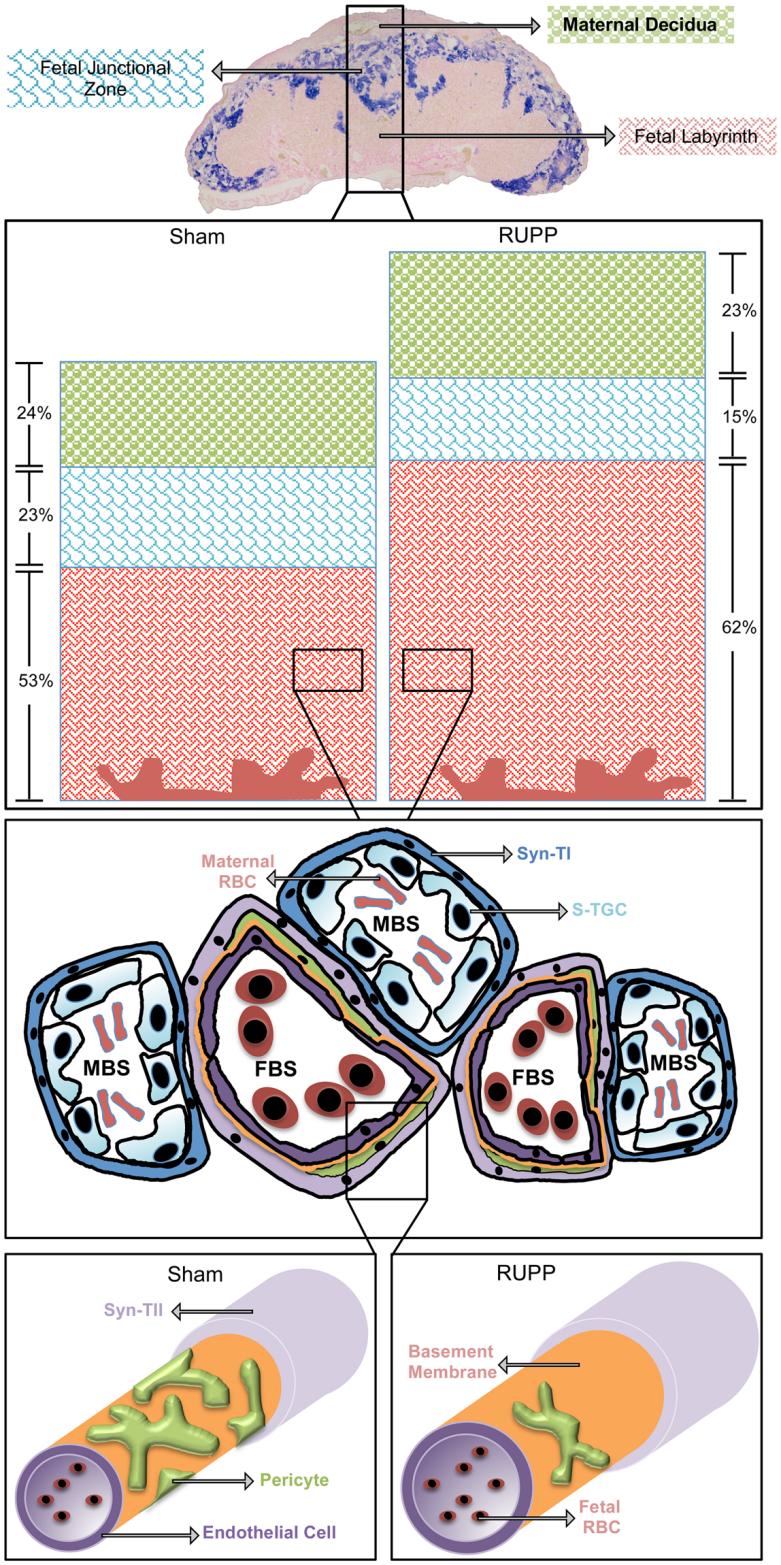
Cartoon represents change in RUPP placentae at E18.5. The 3 placenta layers are visible in the E18.5 placenta with the *Tpbpa* ISH staining in blue identifying the junctional zone, with the maternal decidua above it and the labyrinth layer below it, closest to the fetus. The stacked bar graphs represent the difference in both the size of the placenta and the relative size of the layers at E18.5. A maternal and fetal blood vessel cartoon allow for visualization of the orientation of each of the cell types that make up the maternal and fetal capillary network of the labyrinth layer. Finally, the 3-dimentional cartoon of the Sham and RUPP fetal blood vessel is representative of the increased fetal blood space area seen in response to RUPP and the reduction in pericytes.

We and others have previously shown that RUPP applied to pregnant mice at E14.5 is a useful model for introducing placental insufficiency and “stress” during pregnancy^14,15^. The impact of RUPP on fetal growth is well-described and the molecules considered important in the development of preeclampsia have been evaluated in the context of RUPP. However, a thorough analysis of the mouse placental phenotype and its underlying pathology has not been reported. Here, we undertook an in-depth analysis of the placental phenotype resulting from this model, to gain better insight into the response of the placenta and potential contributors to restricted fetal growth and the onset of maternal symptoms of preeclampsia. So that the results can be easily visualized, a figure representing the results has been created (Fig. 9).

Analysis of fetal and placental weights to assess efficacy and reproducibility of RUPP in in the mouse in our hands included an earlier time point, of 48 hours post-surgery (E16.5) as well as the published E18.5. Interestingly, although RUPP caused a statistically significant decrease in fetal weights by E18.5 and increase in placental weight, there was no detectable difference in either fetal or placental weights at E16.5. These findings were in contrast to many studies using RUPP that report decreased placental weight^4,14,61,62^. However, it is important to note that there is discrepancy in reported placental weight using this model. The same group, Granger *et al*., have reported a non-significant increase, no change and a decrease in placenta weight^7,10,63^ (respectively) in different experiments using the same RUPP model in the rat, and irrespective of placenta weight, fetal growth restriction was observed. While many groups do not report placenta weight at all, increased placenta weight was reported by the Bruce and colleagues^64^. Further, other models of fetal growth restriction in rodent report decreased fetal weight with an increased placental weight, including spontaneously hypertensive rats^65^ and 11bHSD1 inhibition in rats^66^. Although in human pathology and animal models, the vast majority of fetal growth restriction is associated with a reduced placental weight, there are human incidences of fetal growth restriction associated with increased placental weight, and a human study evaluating intrauterine growth and blood pressure in adult life, show that the intrauterine environment has an effect on blood pressure and hypertension in adults. The highest blood pressures occurred in men and women who had been small babies with large placentas. They propose that discordance between placental and fetal size may lead to circulatory adaptation in the fetus, altered arterial structure in the child, and hypertension in the adult^67^. Further supporting this, smoking during pregnancy causes fetal growth restriction with some reports of enlarged placentas with adaptive angiogenesis where peripheral and central chorionic villous tree vascular density increased^68,69^. The smoking studies support altered fetal placental vasculature as one of the contributors to the increased placental weight observed in our study, as the fetal blood space area increased in response to RUPP. Increased fetal blood space together with a non-significant increase in labyrinth size relative to placenta size, increased trophoblast proliferation identified by pHH3 and increased S-TGC populations together, likely account for the increase in placental weight. The significance of the increased placental weight is unknown, and further studies would need to be done to assess whether these pups would be at increased risk of adult hypertension. Our goal in the present study, however, was to ascertain potential contributors to the reduced fetal weight at E18.5. Fetal blood space was altered at E18.5 with increased blood space and decreased perimeter to area ratio. With no change in placental weight or placental layer sizes or ratio of labyrinth to junctional area at E16.5, we evaluated the placenta by cell type in order to ascertain what changes were taking place that led/contributed to the altered fetal/placental weight and fetal blood spaces seen at E18.5.

Proliferation in term human PE and IUGR placentas is well investigated, and it has been suggested that there is no change in trophoblast proliferation^70,71^, when evaluated at term. This supports our observations that in response to RUPP, we see no significant change at E18.5 in proliferation. However, at E16.5 proliferation is altered, with increased pHH3^+^ trophoblast. Low oxygen tension has been shown *in vitro* to trigger trophoblast proliferation^72^. Hypoxia-inducible factor-1 alpha (Hif-1*α*), a regulator of oxygen homeostasis is co-expressed with TGF-β-3 in the developing placenta and as oxygen tension increases, levels of both Hif-1*α* and TGF-β-3 are reduced. It has been shown that inhibition of Hif-1*α* in a hypoxic environment results in the inhibition of TGF-β-3 thereby causing an arrest in trophoblast proliferation. Following this rationale, as TGF-β-3 expression has been shown to be directly regulated by HIF-1^73^, we propose that the hypoxic environment in the RUPP placentae lead to Hif-1*α* mediated expression of TGF-β-3 causing increased trophoblast proliferation. Supporting this, we previously showed that Sca-1^+^ trophoblast and Eomes^+^ trophoblast populations were increased *in vivo* at E16.5 in response to RUPP^15^, and this study identifies increased Epcam at the same time. Taken together, it is possible that increased Hif-1*α* as a result of the hypoxic environment, triggers increased TGF-β-3 expression, creating a microenvironment that promotes increased trophoblast proliferation and markers of TS and progenitor populations. Whether these populations functionally contribute to the RUPP placenta is currently unknown. In culture, TS cells are self-renewing^74–76^, while *in vivo*, different microenvironments may support self-renewal while others may promote division into unequal daughters and/or differentiation. The human TS cell was recently identified^77,78^ and groups are currently investigating them, however their role in the compromised placenta is yet unknown and warrants further investigation.

Given that fetal blood spaces and the fetal to maternal blood space ratio were altered by E18.5 it was key to identify the timing of the changes of the contributing cell populations. Within the labyrinth, placental pericytes were decreased at E16.5 as well as at E18.5, while labyrinth endothelial cells showed increased proliferation at E16.5. It has been suggested that TGF-β-3 may negatively regulate Gcm-1 expression^72^, thus being a potential contributor to the reduction in the relative area of *Gcm-1* expression observed at E16.5 in response to RUPP. In contrast, there was a significant increase in the number of *Ctsq*^+^ S-TGCs at E16.5, which persisted through E18.5. While lineage tracing in response to RUPP would be required to confirm that the pHH3^+^ trophoblast differentiate to S-TGCs, it is not unreasonable to speculate that this may be the case. Supporting this, TS cells in normoxic conditions activate the HIF pathway during differentiation and Arnt null TS cells differentiate primarily to syncytiotrophoblast with a near absence of TGC^55^. Similarly, TS cells derived from HIF-null mice exhibit differentiation defects and fail to form TGCs *in vitro*, suggesting that HIF-1*α* signalling is required for TGC differentiation^54^. Thus, it would stand to reason that increased Hif-1*α* in response to RUPP might preferentially drive differentiation of pHH3^+^ trophoblast to S-TGC. Further supporting this, our TS cell hypoxia studies showed that hypoxia increased *Hif-1α* and promoted differentiation to S-TGCs.

Placental pericytes are derived from the fetal mesoderm and surround fetal endothelial cells as the capillary network forms^79,80^. There is little known about the role of the pericyte in the placenta and how its presence or absence may affect placenta pathology, and the downstream effect on maternal and fetal health. Supporting our finding of reduced placental pericytes, several other groups have reported similar pericyte reduction including: a humanized mouse model hANG x hRN (PAH), with increased maternal blood pressure and reduced fetal weight^81^ and in both Pdgfb and Pdgfrb homozygous knock out mice^80^. These models present with increased maternal blood pressure decreased fetal weights and perinatal mortality. These models, when considered together with the RUPP model (a model with symptoms that mirror preeclampsia, including increased maternal blood pressure and fetal growth restriction), suggest that pericytes are critical to labyrinth development and growth, and their altered presence affects the development of placental vasculature, thereby contributing to fetal growth restriction. However, whether the depleted pericytes contribute to or may be the cause or effect of increased maternal blood pressure is unknown. Studies suggest that the lack of pericyte coverage of the fetal micro vessels lead to enlarged, leaky blood vessels^80^. Thus, it is reasonable to speculate the reduced pericyte coverage is a contributor to the enlarged fetal blood area and reduced fetal weight seen at E18.5 in RUPP. The hypoxic environment generated in the RUPP placenta^14^ may be a contributor to the pericyte phenotype as it has been shown that hypoxia limits pericyte survival/recruitment^82^ in bovine retina studies. The same study showed that Vegfa confers pericyte survival in hypoxic conditions^82^ leading us to identify a novel association of placental pericytes with *Vegfa*^+^ trophoblast in the Sham placenta with reduction in both pericytes and *Vegfa*^+^ trophoblast in response to RUPP, suggesting the relationship may be interdependent. Our TS culture studies showing that labyrinth specific trophoblast *Gcm-1* expression and *Vegfa* expression overlapped, support this idea.

The microarray of E16.5 placentae identified a reduction in the expression of a number of genes, including two collagen genes, prompting evaluation of placental collagen. The relative area of collagen staining was reduced at both E16.5 and E18.5 in the RUPP placentae. Pericytes both re-model and secrete ECM during vessel formation and are influenced by the composition of the ECM through integrin signaling^83–85^. Additionally, pericyte/endothelial cell interactions are important for ECM deposition/remodeling^86^, with trophoblast also contributing to ECM deposition. With increased endothelial proliferation, decreased pericyte numbers, altered *Gcm-1* distribution and increased S-TGC’s, there are multiple potential contributors to the altered collagen and similarly, the reduced collagen may also have impacted the respective cell types/populations.

The mature placenta is composed of a vascular network including maternal blood sinuses and a fetal capillary network, separated by cell layers that facilitate nutrient, gas and waste exchange between the mother and the developing embryo/fetus. This interhemal-membrane is comprised of trophoblast and endothelial cells with trophoblast lining the maternal blood spaces and endothelial cells lining the fetal capillaries. Associated with the endothelial cells, but less often discussed in the context of the placenta, are pericytes. Pericyte-endothelial interaction is essential for the development and function of vascular networks and has been studied in embryonic development, tumorigenesis and tissue remodelling. Interestingly, although complications of pregnancy, including IUGR and preeclampsia, are often associated with “under developed” placentae with abnormalities in the vascular network (labyrinth; mouse or villous tree; human), the role of pericytes in these pathologies, have not been widely investigated. The relationship between pericyte coverage of fetal micro vessels and elevated maternal blood pressure has received little attention, however supporting this concept are the maternal hypertension animal models that have reported decreased placenta pericyte populations. Modeling preeclampsia, hANG x hRN (PAH) mouse pregnancies have elevated maternal blood pressure E12 through term (E19–20), fetal IUGR, maternal proteinuria and convulsion^87^. Micro vessel densities, of PAH fetal vasculature in term placentae, were significantly lower than control placentae, with PAH terminal vessels lacking pericytes and basement membrane, with irregular interaction between the maternal blood sinuses and the fetal micro vessels of the labyrinth at E16. In this model the maternal hypertension precedes the pericyte reduction and the down regulation of Ang-1/Tie-2 between E13 and E16 is implicated as a cause of the decelerated micro vessel maturation, however further investigation is required to unravel the relationship. The RUPP model is the first non-genetic mouse model, displaying the same relationship. As such, using RUPP as a model of pericyte reduction along with genetic models will facilitate the investigation of relationship between placenta pericytes and maternal hypertension, and may contribute to our understanding of placental pathologies. Together we believe this finding warrants further investigation in the context of interdependency between trophoblast and pericytes and the role Vegfa, hypoxia and ECM may play.

The relative size of the junctional zone and area of *Tpbpa* expression was smaller in the RUPP placentae at E18.5 when compared to Sham. Further assessment identified reduced *Tpbpa*^+^ cells in the decidua of the RUPP placentae at the same time, suggesting that the cells had restricted migration potential. ECM is known to affect the migration potential of trophoblast cells^88^, and as discussed above collagen was reduced in the RUPP placentae. Two trophoblast populations migrate to the decidua, the TGCs contribute to spiral artery remodelling and glycogen trophoblast (Gly-T) migrate in the latter half of gestation in murine animals to the decidua, presumably to provide a source of nutrients from the glycogen that they accumulate and store earlier in pregnancy. Human high glycogen distal extra villous trophoblast (EVT) are the most similar to the mouse Gly-T^89^, in that they both store glycogen and both interstitially invade into the maternal decidua, though the mouse Gly-T cell is not as invasive as the human EVT and in the human, glycogen is stored in multiple cell types. Both compromised trophoblast invasion and changes in glycogen deposition are hallmark features of compromised pregnancies in the human^34,90–96^ and in the rodent^34,97–101^. Thus, a mechanism or pathway limiting migration may explain the reduced fetal weight observed in this model. Kisspeptin has been associated with inhibiting cell migration^31^, and is abundantly expressed in the placenta^31^. Evaluation and localization of Kisspeptin expression found the most abundant Kisspeptin staining in the Gly-T. *Aldh1a3* (Gly-T) area of expression was not changed between Sham and RUPP placentae at E16.5, though Kisspeptin staining was increased and histological assessment highlighted more Kiss-1^+^ Gly-T at E16.5. It is possible that migration alone was not the problem; rather, survival in the decidua could be an additional problem, which is supported by the increased presence of *Gzmc*^+^ uNK cells and the increased cell death observed at E16.5.

While many animal studies evaluate placental phenotypes at a single time point, this study highlights the value of assessing gene expression and placental pathology across multiple gestational ages. Sometimes the significance is in the change or lack thereof over time. Further, it identifies populations of cells that are altered early but are corrected by term. This is of significance as populations that are thought unchanged due to their equal representation at E18.5 may have significant effect when altered at an earlier gestational time point. Evaluating expression over multiple time points will also allow investigators to assess more accurately whether expression is actually increased or decreased, rather than the timing of expression having shifted. This does, however, make assessment costly and does increase the number of animals needed; as such the value of this assessment needs to be carefully considered in the context of the model and the hypothesis.

Finally, culture experiments supported the notion that hypoxia may contribute to the trophoblast phenotypes identified in the RUPP placentae. Further studies will be needed to identify whether hypoxia and/or Hif-1*α* contribute to the identified pericyte phenotype.

### RUPP as a model of adaptation

Studies in the last decade have shown that the placenta is not a passive organ that mediates the maternal-fetal exchange; rather, it has the potential to adapt its capacity to supply nutrients in response to both intrinsic and extrinsic stimuli^102^. It has been shown in human pregnancies and animal models that the placenta adapts with morphological changes including, fetal capillaries^103–112^, maternal blood spaces^104,108,109,112^, interhaemal barrier thickness^103,105,106,108,111,113,114^, labyrinth or junctional zone volume^110,112^ 2011 and glycogen cell mass^112,115,116^ in response to various intrinsic and extrinsic factors. Increasing fetal size places an increased level of demand on the placenta and requires an increased level of supply. Reduced fetal size can be the result of reduced supply^117^ or of reduced demand^118^. This study highlights the value of RUPP as a model of reduced supply and an ideal model in which to use genetically engineered mice to evaluate identified populations and their contribution to the adaptive potential of the placenta.

## Materials and Methods

### Animals and cell culture

Placentae were dissected at different stages of gestation from pregnant CD-1 females. A minimum of 5 to 10 dams were dissected at each stage, for each control and experimental group described below. Placentae were fixed in 4% paraformaldehyde overnight, embedded in paraffin and sectioned for analysis by histological staining; immunohistochemistry and *in situ* hybridization as previously described^18,75,119^. RUPP experiment surgeries were conducted as previously described in the rat^4^ but modified to include bilateral ligation of the uterine arteries with surgical thread instead of a surgical clip and no ligation of the abdominal aorta. Sham and non-operated controls, in which no ligation and no surgery were performed respectively, were used as controls. All mouse work was conducted in accordance with University of Calgary and University of California San Diego guidelines for use of animal models in research. Mouse user protocols and experiments were reviewed and approved by the Animal Care Committee at the University of Calgary and the Institutional Animal Care and Use Committee at the University of California San Diego.

Our lab derived the mouse trophoblast stem (TS) cell line from E3.5 blastocyst-staged embryos^15^. Briefly, TS cells were cultured in 5% CO_2_ in humidified air at 37 °C in RPMI culture media supplemented with 20% FBS, 1 mM sodium pyruvate, 50 μg/mL penicillin/streptomycin, 25 ng/mL FGF4, 10 ng/mL recombinant Activin A, 1 μg/mL heparin and 5.5 × 10^−5^ M β-mercaptoethanol as previously described to maintain undifferentiated, proliferating cells^75^. Cells were stimulated to differentiate by the removal of FGF4, activin and heparin.

### Immunostaining

Immunohistochemistry (IHC), was conducted on 5-7 μm tissue sections. Sections were de-paraffinized and rehydrated followed by antigen retrieval in Citra Buffer (Biogenex, USA) using a 2100-Retriever (Electron Microscopy Science, USA). The IHC sections were then treated with 3% H_2_0_2_ to quench endogenous peroxidase, washed in PBS and blocked for 1 hour at room temperature in 1x PBS, 5% goat serum, 0.1% BSA. Sections were incubated at room temperature for 1 hour in primary antibody diluted 1:100 to 1:300 in block solution, based on prior optimization of each antibody. The remainder of the protocol followed the manufacturer’s protocol (Vectastain, Vector Labs, USA). Antibody binding was visualized using Dako DAB according to the manufacturer’s protocol (Dako, USA). Following counterstaining in hematoxylin (Gills #2, Sigma, USA) or nuclear fast red (Vector Labs, USA), sections were dehydrated and mounted in xylene-based mounting medium. The IF sections were de-paraffinized, underwent antigen retrieval and were blocked as for IHC. Sections were then exposed to primary antibody, diluted 1:100 to 1:300, in block and incubated overnight at 4 °C. Following primary antibody incubation, sections were washed in PBS and exposed to Cy5-anti-rabbit or Alexa488-anti rat 2° antibodies, both diluted 1:300 in block, for 1 hour at room temperature followed by PBS wash, counterstaining of nuclei with Hoechst 33342 (1:1000, Molecular Probes, Invitrogen, USA) as previously described^120^. All immunohistochemistry experiments were conducted with their respective negative controls.

### Histological Semi-Quantitative Evaluation

After ISH and/or IHC, slides were photographed with a minimum of 6 placentae per treatment group per gestation were randomly selected. Using Image J, NBT/BCIP or DAB staining was measured relative to the area of the placental section (excluding membranes). A single observer, blinded to the experimental conditions at the time of the assessments, performed all Image J assessment/quantification. The assessments were repeated a second time with a second blinded observer to confirm all results. Each ISH assessment (*Aldh1a3, Ascl2, Ctsq, Gcm-1, Gzmc, Prl3b1 (Pl2), Prl2c2 (Plf), Prl7a7 (Plpf), Rps3a, Tpbpa*, and *Vegfa*) included a measurement of stained area relative to the area of the respective placenta and is presented as a percentage of area stained. Evaluation of *Ctsq* was performed using 40x magnification images and Image J, where individual stained cells were counted within 3 labyrinth specific fields of view/placenta. Number of cells stained is represented as the number of positive cells within the field of view. Ki67 IHC, slides were photographed at 10x magnification and included the chorio-allantoic membrane at the bottom of the image with the remainder of the image including the labyrinth layer. Positive cells were manually counted and were evaluated as a percentage of total nuclei. Ki67/K18, pHH3/K18 and pHH3/Pecam dual IHC stained slides were photographed at 10x magnification and included the chorio-allantoic membrane at the bottom of the image with the remainder of the image including the labyrinth layer. All photos originated at a branch point. Positive or dual positive cells were counted as a total number of dual stained cells within the field of view. All counts included 3 regions/placenta and a minimum of 6 placentae per group. Perforin, TUNEL, TGF-β-3, Epcam and Kisspeptin stained images were photographed at 4x magnification and using Image J positive staining was measured relative to the area of the placental section (excluding membranes). *α*SMA IHC were evaluated at 40x magnification where stained area was measured within 3 labyrinth specific fields of view/placenta, with a minimum of 6 per treatment per gestational age being assessed. Area is presented as a percentage of the field of view. Placenta layers were evaluated, using *Tpbpa* ISH slides. The junctional zone was identified as the *Tpbpa*^+^ cells between the maternal decidua (identified by parietal TGC) and the labyrinth layer and was manually traced. The junctional zone area was presented as a percentage of the total placenta area. Using the same images, the area below the junctional zone was considered the labyrinth layer (including the chorioallantois) and was evaluated as a percentage of the total placenta area, with a minimum of 6 placentae per group assessed. *Tpbpa*^+^ cells in the decidua were used as a measure of trophoblast invasion and migration. Using Image J, the decidua was manually outlined using the P-TGC as the boundary between the junctional zone and the decidua. Once outlined the remainder of the placenta was electronically removed from the image, and *Tpbpa*^+^ staining was quantified as described above, and evaluated relative to the decidua area. Labyrinth blood spaces were assessed using ImageJ where pictures of 3 regions per placenta in a minimum of 6 placentae were taken using a 40x objective. Maternal blood spaces were identified by AP staining and using the same image both maternal and fetal blood spaces were manually outlined, and the area is presented as a percentage of the field of view, while simultaneously measuring the perimeter for each blood space traced, allowing for a total blood space area to total perimeter to be calculated.

### Hypoxia Chamber

Inflatable hypoxic chambers were adapted and modified as previously published^15,121^, using sterile 4.5 mm heat-sealable pouches (VWR, USA). Individual chambers were made to allow for collection of cells after 2, 4, and 6 days and filled with mixed air containing 1% oxygen, 5% CO_2_, and N_2_ balanced (Airgas, USA). Cells were collected for RNA in RNA lysis buffer (Aurum, BioRad, USA), or were fixed in 4% paraformaldehyde (Sigma, USA), for 20 minutes at room temperature.

### RNA isolation and analysis of gene expression

Total RNA was isolated from TS cell cultures using RNeasy or Aurum microspin columns according to the manufacturer’s instructions (Qiagen and BioRad, USA, respectively). Total RNA was isolated from Sham and RUPP placenta using Direct-zol RNA miniprep kit (Zymo Research) according to the manufacturer’s protocol.

mRNA expression was assessed by quantitative **r**everse **t**ranscription (RT)-PCR using the SYBR green method as previously described^25,120^. 1 μg of total RNA was reverse transcribed using the iScript RT kit for SYBR green (BioRad, USA). qRT-PCR reactions were then prepared using the iTaq Universal SYBR green supermix (BioRad, USA), according to the manufacturer’s instructions, and performed on a BioRad CFX96 thermocycler. All primers for qRT-PCR were obtained from BioRad. qRT-PCR reactions were conducted in triplicate on cDNA representing three independent experimental replicates for each gene and data was compiled and analyzed for significant changes in gene expression using the Relative Expression Software Tool (REST) and Ppia and Ywhaz as reference genes^122^.

RNA *in situ hybridization* (ISH) was conducted on 7 μm sections of mouse placenta tissue as previously described^60,75,119^. cRNA probes for *Gcm1, Ctsq, Vegfa, Tpbpa, Prl7a1, Prl2c2* are published^15,29,119^, while *Gzmc* and *Rps3a* probes were transcribed from PCR template generated using primers incorporating T7 and T3 RNA polymerase promoter sequences.

### Microarray

Microarray experiments were conducted in triplicate on E16.5 placentae from independent RUPP or Sham experiments to examine the influence of RUPP on placental gene expression. RNA was isolated from placentae after homogenization in Trizol Reagent (Ambion, USA), using the Direct-zol kit as described above. RNA quality was confirmed using Bioanalyzer and gene expression profiling was performed using Affymetrix mouse, 1.0ST chips (Affymetrix, Santa Clara, CA) and conducted by the Southern Alberta Microarray Facility (University of Calgary, Calgary, Canada). Briefly, cDNA target preparation, labeling, and hybridization were carried out according to standard Affymetrix protocols utilizing Hybridization Oven 640, Fluidics Station 450, and Scanner 3000 (Affymetrix, Santa Clara, CA). Image processing, normalization, and data analysis were performed initially using the Affymetrix Microarray suite. Genes with a false discovery rate P value of less than 0.05 and a signal log ratio absolute fold change of 1.25 were considered significantly different.

Microarray data was assessed for gene set enrichment using Metascape^24^ (http://metascape.org). Gene list, pathway and process enrichment analysis was carried out with the following ontology sources: KEGG Pathway, GO Biological Processes, Reactome Gene Sets and CORUM. All genes in the genome were used as the enrichment background. Terms with p-value < 0.01, minimum count 3, and enrichment factor >1.5 (enrichment factor is the ratio between observed count and the count expected by chance) are collected and grouped into clusters based on their membership similarities. More specifically, p-values are calculated based on accumulative hypergeometric distribution^2^, q-values are calculated using the Banjamini-Hochberg procedure to account for multiple testing^3^. Kappa scores^4^ were used as the similarity metric when performing hierarchical clustering on the enriched terms and then sub-trees with similarity >0.3 are considered a cluster. The most statistically significant term within a cluster is chosen as the one representing the cluster.

### Statistical Analysis

In order to assess whether weight, blood space or gene expression was altered in response to RUPP, two questions were asked: 1) Did RUPP alter expression (multiple comparison analysis identified whether it was universal or specific to a time point), and 2) was expression different at E18.5 when compared to E16.5 (multiple comparison analysis identified whether it was universal or specific to a treatment). Statistical analysis was performed using a two-way ANOVA, followed by Sidak’s Multiple Comparisons Test and significance was reported with a p value ≤ 0.05. When secondary analysis of a particular subgroup was required, statistical analysis was performed using a Paired Student’s t-test, with significance reported with a p value ≤ 0.05. Statistical analysis of ratios between groups was performed using an unpaired t-test with Welch’s correction, with significance reported with a p value ≤ 0.05.

## Electronic supplementary material

Supplementary Figures

Supplemental Table 1
