# Supplementary material for: Reduced Uteroplacental Perfusion Pressure (RUPP) causes altered trophoblast differentiation and pericyte reduction in the mouse placenta labyrinth
Source: Sci Rep. 2018 Nov 21;8:17162. doi: 10.1038/s41598-018-35606-x (PMC6249310; doi:10.1038/s41598-018-35606-x)
Supplement: Supplementary file 1 — Supplementary Figures [file 41598_2018_35606_MOESM1_ESM.pdf]

SUPPLEMENTARY FIGURES

Title: Reduced Uteroplacental Perfusion Pressure (RUPP) causes altered trophoblast differentiation and pericyte reduction in the mouse placenta labyrinth

Authors: Bryony V Natale[2 ♭], Prutha Mehta[2 ♭], Priscilla Vu[2], Christina Schweitzer[1], Katarina Gustin[2], Ramie Kotadia[2] and David R C Natale[1,2]

Affiliations:

[1] Department of Comparative Biology and Experimental Medicine, Faculty of Veterinary Medicine, University of Calgary, Calgary, AB, Canada. T2N4N1.

[2] Department of Obstetrics and Gynecology in Reproductive Sciences, Faculty of Medicine, University of California San Diego, La Jolla, CA, USA. 92093.

Supplemental Figure 1. RUPP does not significantly affect litter size or resorption. Litter size in 15 Sham and 16 RUPP pregnancies was assessed ($n \geq 15$ litters). Resorption sites and dead pups were also assessed. Litter size was not affected. Resorption and dead pups were insignificantly increased in response to RUPP.

Supplemental Figure 2. RUPP does not alter expression of inflammation genes at E16.5 or E18.5. Evaluation of *IL-6* and *TNFa* at E16.5 and E18.5 by qPCR does not identify a significant change in response to RUPP (n=3 placentae per treatment/gestational age).

Supplemental Table 1. RUPP alters gene expression as assessed by microarray. 487 genes were affected ($Fc > 1.25$; $P < 0.05$), with 339 up regulated and 148 down regulated in the RUPP placentae when compared with Sham placentae at E16.5 (n=3 for each treatment group).

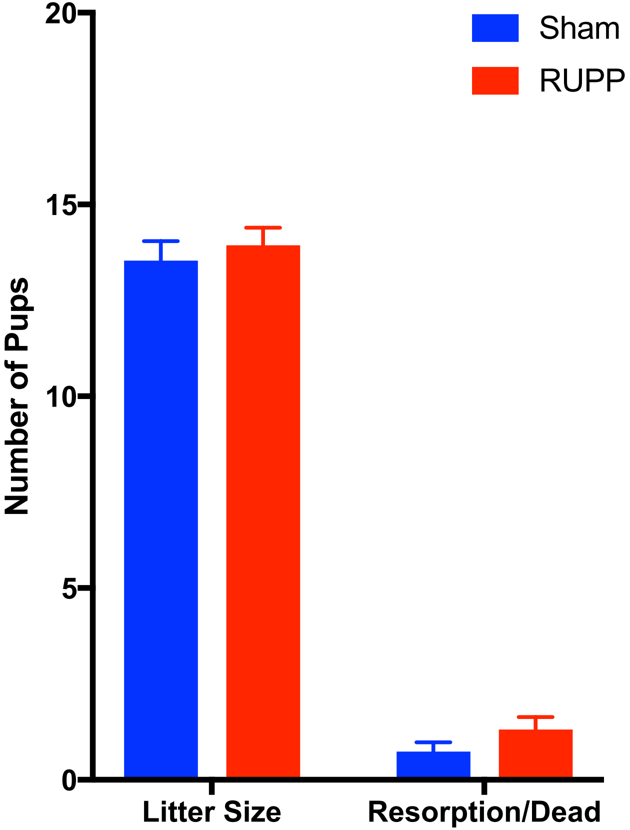
Sham
RUPP
Number of Pups
20
15
10
5
0
Litter Size
Resorption/Dead

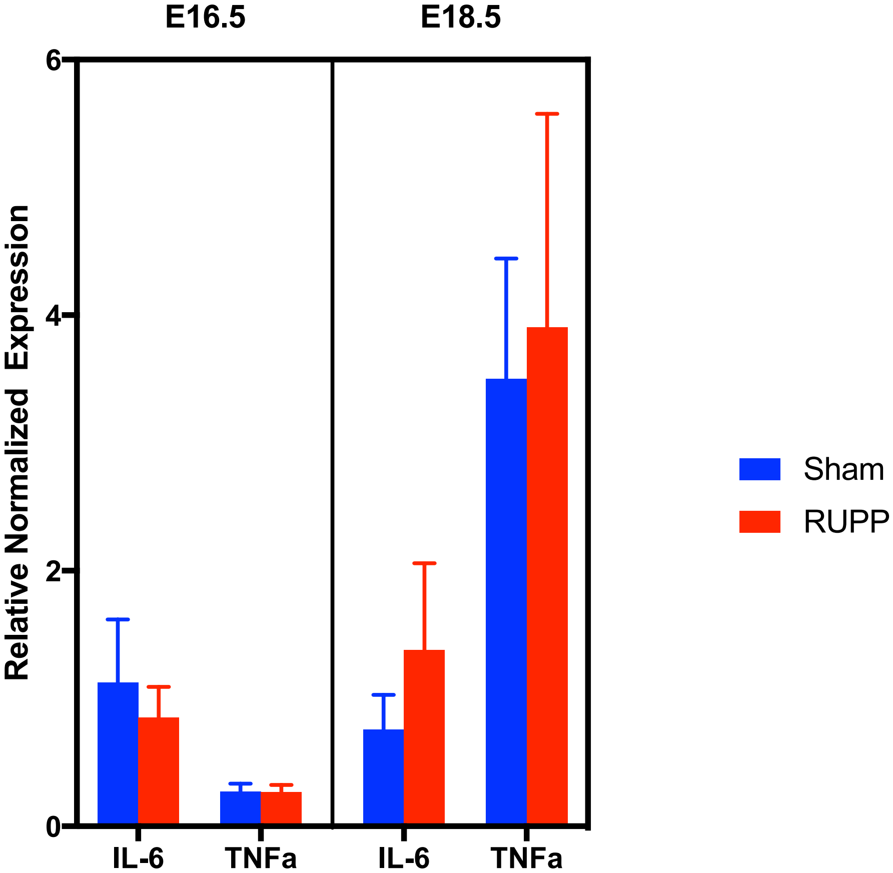

E16.5
E18.5
Relative Normalized Expression
6
4
2
0
IL-6
TNFa
IL-6
TNFa
Sham
RUPP
